# Vigilancia epidemiológica de agentes bacterianos causantes de enfermedades transmitidas por alimentos en el norte de Chile, 2016-2022

**DOI:** 10.15446/rsap.V26n6.111847

**Published:** 2024-11-01

**Authors:** Mirko J. Ortiz-Álvarez, Edgardo R. Santander-Pulgar, Alejandra K. Allendes-Siles, Marcelo A. Vicencio-Ahumada

**Affiliations:** 1 MO: Ing. Biotec. M. Sc. Diagnostico Microbiológico. Universidad Arturo Prat. Iquique, Chile. mirkoortiz@hotmail.cl Universidad Arturo Prat Universidad Arturo Prat Iquique Chile; 2 ES: BM. M. Sc. Ciencias Biológicas. Universidad Arturo Prat. Iquique, Chile. esantan@unap.cl Universidad Arturo Prat Ciencias Biológicas Universidad Arturo Prat Iquique Chile esantan@unap.cl; 3 AA: TM. Universidad de Tarapacá. Arica, Chile. ale.allendes.siles@gmail.com Universidad de Tarapacá Universidad de Tarapacá Arica Chile; 4 MV: Ing. Alim. Universidad de Chile. Calera, Chile. m_vicencioa@hotmail.com Universidad de Chile Universidad de Chile Calera Chile

**Keywords:** ETAs, vigilancia, agentes bacterianos, epidemiología, Tarapacá, Chile *(fuente: DeCS, BIREME)*, FBDs, surveillance, bacterial agents, epidemiological, Tarapacá, Chile *(source: MeSH, NLM)*

## Abstract

**Objetivo:**

Describir las bacterias notificadas en matrices relacionadas con las enfermedades transmitidas por alimentos (ETAs) en la región de Tarapacá (Chile).

**Materiales y Métodos:**

Se llevó a cabo un estudio descriptivo del total de las notificaciones informadas por el sistema vigilancia en Tarapacá, desde el año 2016 hasta el 2022. Las variables analizadas fueron bacteria causal, alimento involucrado, programa de vigilancia y distribución temporal de las bacterias notificadas durante dicho periodo.

**Resultados:**

Durante el periodo analizado se notificaron 184 cepas de diferentes bacterias *(Salmonella spp, Staphylococcus aureus, Listeria monocytogenes, Vibrio para-haemolyticus, Vibrio cholerae* y *Bacillus cereus).* Los productos cárnicos y los platos preparados, en conjunto con los productos de la pesca son los que contienen más detecciones bacterianas. El programa de vigilancia regional de alimentos ha notificado todas las bacterias mencionadas y es el que tiene mayores notificaciones, seguido de las importaciones. Se notificaron más bacterias en alimentos que en agua. El año 2019 fue el que tuvo un mayor número de bacterias notificadas (68 cepas). *Salmonella spp* y *Staphylococcus aureus* fueron las bacterias que se notificaron todos los años. *Salmonella spp* y *Vibrio parahemolyticus* fueron las que tuvieron un mayor número de notificaciones, con 34,8% y 22,8%, respectivamente.

**Conclusión:**

La información solicitada a través de transparencia activa a la Secretaría Regional Ministerial de Salud de Tarapacá y al Instituto de Salud Pública permitió identificar las bacterias con mayor ocurrencia y más frecuentes. También se logró identificar que los programas de vigilancia regional y de importaciones deben fortalecerse y que los productos cárnicos, en conjunto con los productos de la pesca, demandan mayor fiscalización.

## Abstract (en)

**Objective:**

To describe the bacteria reported in matrices related to foodborne diseases (FBDs) in the Tarapacá region (Chile).

**Materials and Methods:**

A descriptive study was conducted on the total number of notifications reported by the surveillance system in Tarapacá from 2016 to 2022. The analyzed variables included the causative bacteria, the implicated food, the surveillance program, and the temporal distribution of the reported bacteria during this period.

**Results:**

During the analyzed period, 184 strains of different bacteria *(Salmonella spp., Staphylococcus aureus, Listeria monocytogenes, Vibrio parahaemolyticus, Vibrio cholerae,* and *Bacillus cereus)* were reported. Meat products and prepared dishes, together with seafood products, were the most frequently detected with bacterial contamination. The regional food surveillance program reported all the aforementioned bacteria and had the highest number of notifications, followed by imports. More bacteria were reported in food than in water. 2019 had the highest number of reported bacteria (68 strains). *Salmonella spp.* and *Staphylococcus aureus* were reported every year. *Salmonella spp.* and Vibrio parahaemolyticus had the highest number of notifications, 34.8% and 22.8%, respectively. During the period analyzed 184 strains of different bacteria (Salmonella spp, *Staphylococcus aureus, Listeria monocytogenes, Vibrio parahaemolyticus, Vibrio cholerae* and *Bacillus cereus)* were reported. Meat products and prepared dishes, together with fishery products, contain the most bacterial detections. The regional food surveillance program has reported all the above-mentioned bacteria and has the most notifications, followed by imports. More bacteria were reported in food than in water. The year 2019 had the highest number of bacteria reported (68 strains). Salmonella spp and *Staphylococcus* aureus were the bacteria reported every year. *Salmonella spp* and *Vibrio parahemolyticus* had the highest number of notifications, with 34.8% and 22.8%, respectively.

**Conclusion:**

The information obtained through active transparency from the Regional Ministerial Secretariat of Health of Tarapacá and the Public Health Institute allowed for the identification of the most prevalent and frequent bacteria. It was also determined that the regional and import surveillance programs need to be strengthened, and that meat products, along with seafood products, require closer monitoring.

Los agentes patógenos se han adaptado con éxito a los avances en las técnicas de producción, procesamiento y conservación de alimentos y aguas. Estos microorganismos, al no ser detectados antes de ingerirlos, causan enfermedades transmitidas por alimentos (ETAs), las cuales representan una amenaza pública latente y han sido notificadas en todo el mundo, con independencia del grado de desarrollo de los países. Lo anterior conlleva graves repercusiones en la salud pública y en el ámbito económico 1. Las ETAs causan síntomas como náuseas y vómitos, dolor abdominal, fiebre, deshidratación, heces sanguinolentas, parálisis, espasmo, hipertensión arterial y diarrea. Esta última es el síntoma más común de todos, y en algunos casos puede incluso terminar en la defunción 2,3. Las ETAs constituyen uno de los problemas de salud pública de mayor importancia a escala mundial, pues conllevan alta morbilidad y mortalidad. Se presentan con mayor frecuencia en poblaciones de bajos recursos y afectan, en general, a todos los estratos etarios y de género.

En Chile, el Ministerio de Salud Pública (MINSAL) financia y administra los programas de vigilancia de alimentos y aguas (potables, piscinas, aguas de mar y plantas de tratamientos), con el objetivo de detectar estos agentes patógenos y así poder controlar la inocuidad y la calidad de los alimentos y las aguas, previniendo así amenazas microbiológicas que puedan perjudicar a la población. En las regiones de Chile, las Secretarías Regionales Ministeriales de Salud (SEREMIS de Salud) ejecuta estas vigilancias por medio de los laboratorios de salud pública ambiental, siguiendo las instrucciones del Reglamento Sanitario de Alimentos (RSA), el cual determina qué microorganismos deben ser identificados en cada matriz. En el caso de las aquellas relacionadas con aguas, los parámetros por los cuales se rigen son los del programa de saneamiento básico de la Superintendencia de Servicios Sanitarios (SISS); y con relación las aguas recreacionales existen el Decreto Supremo 144.

En Tarapacá, entre el 2016 y el verano del 2022, la SEREMI de Salud, a través del laboratorio de salud pública ambiental, llevó a cabo el seguimiento de microorganismos (bacterias) en las matrices antes mencionadas. Posteriormente al diagnóstico, las cepas aisladas fueron remitidas al laboratorio nacional y de referencia del Instituto de Salud Pública (ISP), para que a través de sus protocolos se realice el estudio de serotipo y confirmación de toxinas para las cepas aisladas. Estas últimas, una vez son confirmadas y clasificadas por el ISP, se notifican, según lo señalado en el decreto N.° 7/20, "Reglamento sobre notificación de enfermedades transmisibles de declaración obligatoria y su vigilancia", en el cual se señala la notificación obligatoria e inmediata de todos los brotes de ETAs, incluyéndose los agentes aislados de muestras ambientales.

Expuesto lo anterior, y derivado de ello, el objetivo de este trabajo es describir la ocurrencia de brotes de bacterias patógenas asociadas a alimentos y aguas en las diferentes matrices relacionadas con el seguimiento de ETAs en la ciudad de Iquique, región de Tarapacá (Chile), reconociendo qué microorganismos tienen una mayor incidencia y cuáles matrices fueron las que presentaron una mayor prevalencia. Esta información permitirá ajustar las políticas públicas del seguimiento de estas bacterias.

## MATERIALES Y MÉTODOS

Se realizó un estudio observacional de tipo descriptivo, inserto en una investigación operacional en el interior del sistema de vigilancia de las ETAs. La sustancia de estudio estuvo conformada por todas las notificaciones de agentes bacterianos en la región de Tarapacá a través del sistema automatizado de vigilancia de ETAs, dependiente del Departamento de Estadísticas e Información de Salud (DEIS) del MINSAL, entre el año 2016 y el verano del 2022. La descripción de la información se basó en el aspecto etiológico bacteria causal, alimento involucrado, programa de vigilancia y distribución temporal de las bacterias notificadas durante dicho periodo.

La base de datos con los registros de brotes notificados fue solicitada al laboratorio de salud pública ambiental de Tarapacá, perteneciente a la SEREMI, y también al laboratorio nacional y de referencia del ISP para cruce de datos, se analizó utilizando el paquete Excel. La información resultante del análisis fue expresada en frecuencias absolutas y porcentajes, y se representó en tablas y gráficos. También se hizo un análisis sistemático bibliográfico que describe las principales bacterias causantes de ETAs. Se incluyó la búsqueda de investigaciones propias del tema, en los idiomas inglés y español, así como documentos de divulgación y conferencias científicas. Como herramienta se utilizó artículos relacionados en las bases de datos de *Web of Science* (WOS) y Scopus, con el término de enfermedades transmitidas por alimentos (ETAS).

## RESULTADOS

Los alimentos son vectores de diversos microorganismos que desencadenan enfermedades y malestares para la salud. En la mayoría de los casos los originan agentes externos, cuando esto sucede se define como contaminación. Estos agentes externos pueden ser físicos, químicos o biológicos.

En este último caso, la contaminación es por microorganismos (i. e., bacterias, mohos, virus y parásitos animales). La contaminación sucede directa e indirectamente. La primera es producida por el manipulador, dado que el cuerpo de este es un hábitat adecuado para la supervivencia de estos gérmenes, y se acumulan en manos, boca, nariz y tracto digestivo. En cambio, la contaminación indirecta es producida a través de insectos, parásitos, agua, utensilios y basura (vectores y fómites).

Los agentes biológicos que causan contaminación en alimentos, como las bacterias, pueden tener efectos adversos para la salud. Algunas bacterias producen esporas como mecanismo de supervivencia a condiciones adversas, y al crecer en número en los alimentos pueden producir toxinas, que afectan su aspecto, color y textura, y que al ingerirlas ocasionan intoxicaciones, infecciones y toxiinfección alimentaria.

El diagnóstico de los agentes patógenos en el seguimiento de enfermedades transmitidas por alimentos consta de metodologías tradicionales como, por ejemplo, el número más probable (NMP), donde se identifica principalmente *Staphylococcus aureus (S. aureus), Bacillus cereus (B. cereus), Vibrio parahaemolyticus (V. parahaemolyticus).* También se emplean métodos que utilizan equipos automatizados, como lo es en el caso de *Salmonella spp* y *Listeria monocytogenes (L. monocytogenes).* En el caso del *Vibrio cholerae,* detectado en matrices de agua, normalmente se encuentra en bajos niveles, por lo que se utilizan técnicas de concentración, como el método de torula de Moore, para la detección de este y otros patógenos entéricos (Tabla 1).

**Tabla 1:** Agentes bacterianos notificados, métodos y referencias del sistema de salud chileno

Bacterias	Laboratorio de Salud Pública Ambiental de Tarapacá		Instituto de Salud Pública de Chile	
	Diagnóstico	Referencia	Método de confirmación	Código interno
*Salmonella spp*	Detección *de Salmonella* por VIDAS UP (SPT)	Método certificado NF VALIDATION BIO-12/32-10/11	Cultivo convencional, reacción en cadena de la polimerasa (PCR), serología	2110037/2110079/2121007
*Staphylococcus aureus*	Determinación de *Staphylococcus aureus* por número más probable (NMP)	U.S. Food and Drug Administration Bacteriological Analytical Manual, BAM Online, Chapter 12. *Staphylococcus aureus*	Cultivo convencional, espectrometría de masa Malditoff, reacción en cadena de la polimerasa (PCR) para determinar factores de virulencia	2110077/2127033/2127055
*Listeria monocytogenes*	Detección de *Listeria monocytogenes* por VIDAS II (LMO2)	Método certificado NF VALIDATION BIO-12/11-03/04	Cultivo convencional, reacción en cadena de la polimerasa (PCR) para determinar factores de virulencia	2127043/2110079
*Vibrio parahaemolyticus*	Determinación de *Vibrio parahaemolyticus* por número más probable (NMP), agua y alimentos	U.S. Food and Drug Administration Bacteriological Analytical Manual, BAM Online, Chapter 9. *Vibrio*	Cultivo convencional, reacción en cadena de la polimerasa (PCR), serología	2110037/2110071/2127048/21270 56/2127057
*Vibrio cholerae*	Determinar la presencia de *Vibrio cholerae* en aguas servidas, por concentración en tórula de Moore	U.S. Food and Drug Administration Bacteriological Analytical Manual, BAM Online, Chapter 9. *Vibrio* -Norma ISO 21872-1	Cultivo convencional, reacción en cadena de la polimerasa (PCR), serología	2110037/2110071/2127036/2127045/2127048
*Bacillus cereus*	Determinación de *Bacillus cereus* por número más probable (NMP)	U.S. Food and Drug Administration Bacteriological Analytical Manual, BAM Online, Chapter 14. *Bacillus cereus*	Cultivo convencional, reacción en cadena de la polimerasa (PCR) para determinar factores de virulencia	2127006

Entre el 2016 y el verano del 2022 se notificaron en el sistema de vigilancia de la Región de Tarapacá, 184 cepas bacterianas patógenas provenientes de distintas matrices de alimentos y aguas. Entre el 2019 y el 2020 se registró el mayor número de cepas, al confirmarse 68 y 37, respectivamente, mientras que el 2016, el 2017 y el 2018 presentaron un número menor a 25 cepas (25, 13 y 19 cepas, respectivamente). En los demás años, el 2021 y el verano del 2022, las notificaciones de estas bacterias variaron entre 13 y 9 cepas.

**Figura 1:**
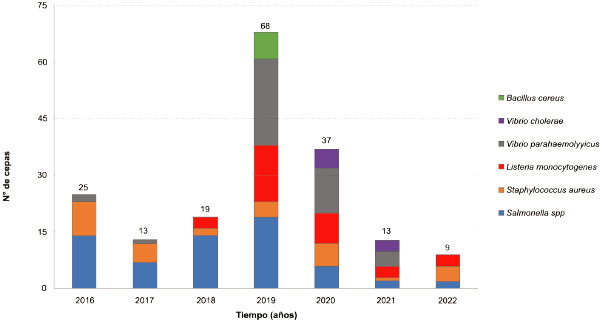
Distribución de agentes bacterianos notificados por año en la región de Tarapacá-Chile, 2016-2022

Al analizar el número de cepas por cada especie bacteriana detectada (Figura 1), dos ocurrieron de forma constante, la *Salmonella spp* y el *Staphylococcus aureus,* mientras que con una menor frecuencia se presentaron *Listeria monocytogenes* y *Vibrio parahaemolyticus.* Por último, con la menor ocurrencia, se presentaron *Vibrio cholerae* y *Bacillus cereus.*

Con referencia a *Salmonella spp,* su mayor número se detectó en el 2019, con 19 cepas notificadas, mientras que en el tiempo restante de estudio se detectaron seis o más cepas, exceptuando el 2021 y el verano del 2022, cuando se detectaron dos cepas. Entre el 2016 y el verano del 2022 se notificaron 64 cepas de esta bacteria.

El *Staphylococcus aureus* fue detectado durante todo el periodo de estudio, pero el mayor evento en número de cepas notificadas fue el 2016, con nueve cepas. Después de este evento disminuyó constantemente, hasta llegar a solo dos cepas notificadas en el 2018. Sin embargo, en el 2019 y en el 2020 aumentó a cuatro y seis cepas, respectivamente. El 2021 fue el periodo con menor número de cepas notificadas, con solo una, mientras que en el 2022 se notificaron dos cepas. Durante todo el tiempo de estudio se notificaron en total 31 cepas de esta bacteria.

*Listeria monocytogenes* fue notificada en el 2018, con tres cepas, y en el 2019 con 15; este último fue su mayor número de notificaciones, y disminuyó progresivamente a ocho cepas el 2020, mientras que en el 2021 y en el verano del 2022 se mantuvo constante en dos cepas. Se notificaron un total de 32 cepas entre el 2016 y el verano del 2022.

*Vibrio parahaemolyticus* no fue notificada en el 2018, ni en el verano del 2022. Entre el 2016 y el 2017 se notificó con solamente tres y dos cepas, siendo este último año aquel con el menor número notificaciones. En el 2019 se notificaron 23 cepas, para después disminuir en el 2020 a 12 cepas y en el 2021 a 8 cepas. Las notificaciones de esta bacteria en total fueron de 42 cepas para todo el periodo de estudio.

*Vibrio cholerae* se notificó solamente en dos años: el 2020 con cinco cepas y el 2021 con tres cepas, con un total de ocho cepas entre el año 2016 y el verano del 2022.

De *Bacillus cereus* solo se notificaron siete cepas, todas ellas en el año 2019.

**Figura 2:**
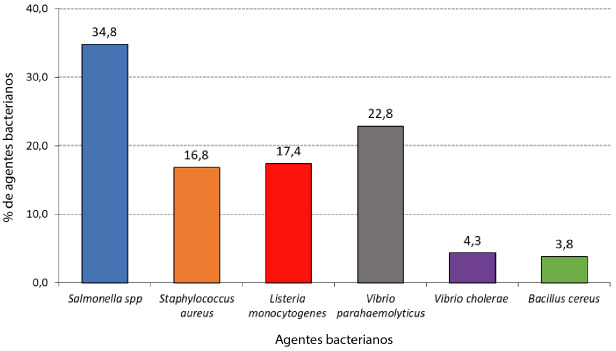
Porcentajes totales de agentes bacterianos notificados en la región Tarapacá-Chile, 2016- 2022

En términos porcentuales, destaca *Salmonella spp* con un 34,8% (64 cepas), a la que le siguen, con un 22,8% (42 cepas), *Vibrio parahaemolyticus; Listeria monocytogenes* con 17,4% (32 cepas); *Staphylococcus aureus* con 16,8% (31 cepas); y *Vibrio cholerae* y *Bacillus cereus,* con 4,3% y 3,8% (8 y 7 cepas notificadas, respectivamente) (Figura 2).

Para realizar las vigilancias de estas bacterias se emplean diferentes programas, dentro de los que se encuentra la vigilancia regional de alimentos, la cual se dedica a detectar bacterias a escala regional, dependiendo de la temporada, y con una búsqueda dirigida a los alimentos más abundantes en las estaciones del año. Un ejemplo de esto es focalizar las vigilancias en mariscos, pescado y alimentos preparados en Semana Santa; otro ejemplo es cuando ingresan los estudiantes a los diferentes establecimientos educacionales, centrar la vigilancia en las leches y sediles.

En este estudio, el programa de vigilancia regional encabeza la notificación de todas las bacterias. Aquella con mayor notificación fue *Vibrio parahaemolyticus,* con 39 cepas, a la que le siguen *Listeria monocytogenes,* con 32 cepas; *Staphylococcus aureus,* con 29 cepas; *Bacillus cereus,* con siete cepas; *Vibrio cholerae,* con cinco cepas; y, finalmente, *Salmonella spp.,* con tres cepas. La suma de todas estas bacterias fue de 115 cepas, que representaron un 62,5%.

Con respecto a la información generada por el programa de vigilancia de importación, el cual se realiza en alimentos y bebidas carbonatadas que ingresan a la Zona Franca de Iquique (ZOFRI), provenientes de distintos países, en este programa se notificaron 47 cepas de la *Salmonella spp.,* correspondientes al 25,5 %.

Por otra parte, el Programa de Vigilancia Nacional de Patógenos se dedica especialmente a los microorganismos de *Salmonella spp* y *Listeria monocytogenes,* en diferentes matrices de alimentos y aguas. De la misma manera que en el de importación, solamente se notificaron *Salmonella spp* (14 cepas), correspondiente al 7,6%. También existe una vigilancia que se especializa en búsqueda de *Vibrio parahaemolyticus;* esta representa el 1,6%, con tres cepas, al igual que el Programa de Saneamiento Básico (aguas de regadío aguas servidas, etc.), que también detectó tres cepas de *Vibrio cholera.*

Otro programa de vigilancia de poca incidencia, pero de no menor importancia son las denuncias por intoxicación de alimentos, que pueden traer como consecuencia ETAs. En este caso, solamente se notificaron dos cepas de *Staphylococcus aureus,* correspondientes al 1,1% (Tabla 2).

**Tabla 2:** Agentes bacterianos notificados en diferentes programas de vigilancias en la región de Tarapacá-Chile, 2016-2022

Programa	*Salmonella spp*	*Staphylococcus aureus*	*Listeria monocytogenes*	*Vibrio parahaemolyticus*	*Vibrio cholerae*	*Bacillus cereus*	Total	%
Vigilancia regional	3	29	32	39	5	7	115	62,5
Vigilancia importación	47	0	0	0	0	0	47	25,5
Vigilancia nacional	14	0	0	0	0	0	14	7,6
Saneamiento básico	0	0	0	0	3	0	3	1,6
Vigilancia *Vibrio parahaemolyticus*	0	0	0	3	0	0	3	1,6
Denuncias	0	2	0	0	0	0	2	1,1
Total	64	31	32	42	8	7	184	100

La matriz con mayores cepas notificadas fueron los productos cárneos, con 34,8%, correspondientes a 64 cepas de dos especies de bacterias, de las cuales 62 corresponden a *Salmonella spp.* Se detectaron 46 cepas en pollo, 12 en carne de ave y cuatro en pavo, mientras que las dos cepas restantes son de *Listeria monocytogenes,* que se encontraron una en pollo y otra en carne de vacuno. Con el 23,9 % y un total de 44 cepas se asociaron a la matriz comida y platos preparados, comida y platos preelaborados que necesitan cocción. Las bacterias notificadas en esta matriz corresponden a 19 cepas de *Staphylococcus aureus,* encontradas en ensaladas (cinco), ensalada de poroto, repollo y lechuga (una) y pescado, cilandro y cebolla (una), palta pelada (cinco), hamburguesa (una), roll de pollo (dos) y lechuga picada (cuatro). También se encontraron 20 cepas de *Listeria monocytogenes,* 18 en ceviche, una en ensalada y otra en sushi. Además, se notificaron cinco cepas de *Vibrio parahaemolyticus* en platos preparados (comida).

En la matriz de pescado y mariscos se notificaron 37 cepas en *Vibrio parahaemolyticus,* correspondiente al 20,1%.

Estas notificaciones fueron 19 cepas en almejas, seis en cholga, 11 en choro y una en mariscos. Las frutas y verduras corresponden al 6,5 %, con 12 cepas notificadas: siete de *Staphylococcus aureus,* notificadas en jugo y pulpa de mango, y las cinco restantes de Listeria monocytogenes, encontrada en pulpa de mango. El 4,9% corresponde a nueve cepas que se notificaron en la categoría de leche y productos lácteos, con siete cepas de *Bacillus cereus* y dos de *Staphylococcus aureus,* en mamaderas (preparaciones reconstituidas). Con el 4,3% y un total de ocho cepas se encontró *Vibrio cholerae* en la matriz de aguas saneamiento básico. Las bacterias notificadas en este último caso corresponden a dos en agua servidas, dos en agua de regadío y cuatro en aguaLos cereales representan el 3,8%, con siete cepas detectadas, correspondientes a dos cepas de *Salmonella spp* notificadas en harina de pescado y cinco cepas de *Listeria monocytogenes* notificadas en fideos. Por último, se encontraron tres cepas de *Staphylococcus aureus,* correspondiente al 1,6%, en helados (Tabla 3).

**Tabla 3:** Agentes bacterianos notificados en diferentes matrices de alimentos y agua en la región de Tarapacá-Chile, 2016-2022

Matriz	*Salmonella spp*	*Staphylococcus aureus*	*Listeria monocytogenes*	*Vibrio parahaemolyticus*	*Vibrio cholerae*	*Bacillus cereus*	Total	%
Productos cárnicos	62	0	2	0	0	0	64	35,5
Platos preparados	0	19	20	5	0	0	44	23,9
Pescados y moluscos	0	0	0	37	0	0	37	20,1
Frutas y verduras	0	7	5	0	0	0	12	6,5
Leches y productos lácteos	0	2	0	0	0	7	9	4,9
Saneamiento básico	0	0	0	0	8	0	8	4,3
Cereales	2	0	5	0	0	0	7	3,8
Helados	0	3	0	0	0	0	3	1,6
Total	64	31	32	42	8	7	184	100

## DISCUSIÓN

Todos los programas de alimentos se rigen por el "Reglamento Sanitario de Alimentos" (RSA) y en el caso de las matrices de aguas por el compendio normativo de la Superintendencia de Servicios Sanitarios (SISS) y de agua potable. A la vez estos, se ajustan a otras leyes, como en el caso del agua de uso recreacional, reguladas por el Decreto Supremo (DS) N.° 144 y el DS N.° 209, que establecen normas de calidad primaria para la protección de las aguas marinas y estuarinas aptas para actividades de recreación con contacto directo. También influyen el decreto (DTO) N.° 977/96, el cual aprueba el reglamento sanitario de los alimentos, y el DS N.° 7, que establece el reglamento sobre notificación de enfermedades transmisibles de declaración obligatoria y su vigilancia.

Al analizar las diferentes matrices donde fue detectada *Salmonella spp,* se notifica y observa su presencia principalmente en productos cárnicos y en cereales, lo que concuerda con otros registros de ETAs producidas por esta bacteria en otros lugares del mundo. Así, en Costa Rica se notificó *Salmonella spp* en productos cárnicos (pollos) y harina de carne y huesos, además, en México se detectó su presencia en productos cárnicos, al igual que en la presente investigación. Sin embargo, se encontraron cepas de *salmonella spp* en las verduras del mercado de La Parada en Perú, y en Estados Unidos se detectó contaminación ambiental en la planta de fabricación que produjo maní Brands X e Y (mantequilla de maní). En Alemania, en chocolate; en España, en ovoproductos, productos cárnicos, crustáceos, pescado, pastelería o confitería; y en Colombia, en huevos de gallina 4-9. El género *Salmonella* forma parte de la familia *Enterobacteriaceae* y es uno de los principales culpables de infección transmitida por alimentos. Las bacterias del género *Salmonella* son Gram negativas, anaerobios facultativos, no formadores de esporas, generalmente móviles con flagelos perítricos (excepto *S. gallinarum),* catalasa positiva, oxidasa negativa y reducen nitratos a nitritos. Asimismo, fermentan glucosa, maltosa y manitol, pero no lactosa ni sacarosa 10. Quizá, su alta prevalencia se deba a que son viables en diferentes condiciones ambientales, sobreviven a la refrigeración y congelación y mueren por altas temperaturas (> 70° C).

Se han reportados brotes de *Staphylococcus aureu*s ocasionados por una deficiente manipulación e higiene del personal en Argentina, Chile y Paraguay. También se detectó esta bacteria en embutidos fermentados en España, en chorizos y longanizas en México y en salame y jamón en El Salvador. Al igual que en el presente estudio, se detectó en platos de comida fría, en funcionarios que eran portadores asintomáticos, además, en Francia se reportaron casos en frutas y verduras, leches y productos lácteos, y en Colombia en carne de res 11-18. El *Staphylococcus aureus* es por mucho el patógeno humano más importante entre los *Staphylococcus.* Se encuentra en el medioambiente externo y coloniza a los mamíferos. Se caracteriza porque se divide en agrupaciones que asemejan a racimos de uva. Son bacterias Gram positivas, no móviles, no esporuladas, sin cápsula, aunque existen algunas cepas que desarrollan una cápsula de limo. Además, son anaerobias facultativas. La mayoría de los *estafilococos* produce catalasa, característica que se utiliza para diferenciar el género *Staphylococcu*s de los géneros *Streptococcus* y *Enterococcus,* que son catalasa negativos 19.

La *Listeria monocytogenes* comparada con otras bacterias patógenas que no producen esporas y que son transmitidas por alimentos, presenta la particularidad de resistir distintas condiciones de estrés como congelación, secado, acidez y frío, pudiéndose adaptar a estas mediante la producción de biofilms. *Listeria monocytogenes* ha sido encontrada con frecuencia en quesos, en países como Costa Rica, Honduras y Ecuador, y en fiambres en Argentina. Al igual que en el presente estudio, se notificó en productos cárnicos en Turquía, en carne y pollo, mientras que en España se encontró en cerdo y también en frutas y verduras, al igual que en Venezuela y Colombia 20-27. Por otra parte, la *Listeria monocytogenes* causa dos formas de listeriosis: gastrointestinal no invasora y listeriosis invasiva. En individuos inmunocompetentes, la listeriosis no invasiva se presenta como un cuadro típico de gastroenteritis febril (fiebre y diarrea) que puede comprometer diversos órganos, con particular predilección por el sistema nervioso central. En pacientes inmunosuprimidos, se puede manifestar como un cuadro de septicemia o meningoencefalitis, con presencia de cefalea, fiebre y somnolencia.

El *Vibrio parahaemolyticus,* que se encontró en ostiones congelados en Uruguay, tiene una alta prevalencia en pescados y maricos, donde se detectó en Perú, mientras que en Tailandia se encontró en ostras, en China en crustáceos y carne de res. En México aparte de *Vibrio parahaemolyticus* en camarón, también se detectó *Vibrio cholerae;* de este último también se encuentran notificaciones en Chile en el 2018 28-33.

*Bacillus cereus* se encontró en arroz en México, Colombia y Arabia Saudita, en ensaladas de pasta en Bélgica, en productos cárnicos en India y Estados Unidos, y en productos de leche y lácteos (productos infantiles) en Irán, al igual que en este estudio 34-39. *Bacillus cereus* es una bacteria perteneciente al género *Bacillus,* capaz de producir toxinas. A pesar de haber sido estudiada con frecuencia, aún no es conocida del todo, y algunos aspectos permanecen por descubrir. Puede estar de forma individual o formando cadenas cortas. Presenta flagelos distribuidos uniformemente por toda su superficie. Sus esporas se caracterizan por ser altamente resistentes a cambios ambientales y a factores físicos como la radiación gamma. Es una bacteria catalasa positiva, con capacidad de producir hemólisis en los eritrocitos. Dependiendo de las condiciones ambientales y de la disponibilidad de nutrientes, puede fermentar varios tipos de compuestos, entre los que se pueden mencionar la glucosa, el glicerol, la salicina y la sacarosa. Además de esto, es capaz de metabolizar nitratos y transformarlos en nitritos. Se ha logrado aislar ejemplares de *Bacillus cereus* en distintos ambientes, como el suelo, el agua, algunas plantas e incluso en el intestino de algunos animales 40. De ello se puede deducir que esta bacteria es capaz de sobrevivir bajo condiciones muy diversas y amplias.

En conclusión, la información solicitada a través de transparencia activa al SEREMI de Salud de Tarapacá y al ISP permitió identificar las bacterias con mayor ocurrencia y más frecuentes. También, se logró identificar que los programa de vigilancia regional y de importaciones se deben fortalecer, y que los productos cárnicos y los platos preparados, en conjunto con los productos de la pesca, demandan mayor fiscalización.

Según los resultados de este estudio, se recomienda establecer controles preventivos para las importaciones antes de la llegada de los productos cárnicos a Chile por la ZOFRI, con el fin de disminuir el ingreso de alimentos contaminados con *Salmonella spp,* también de educar a pescadores artesanales como industriales sobre *Vibrio parahemolyticus* y sus consecuencias, ya Iquique está junto al mar, y se trata de una práctica cotidiana y motor de economía local; además, realizar campañas de difusión sobre los agentes bacterianos que producen las ETAs y los peligros que estas conllevan, y por último aumentar la fiscalización de eventos masivos donde concurra la población, como lo son las fiestas religiosas de Tarapacá y de La Tirana ♣

## References

[1] Jiménez-Edeza M, Castillo-Burgos M, Germán-Báez LJ, Castañeda-Ruelas GM (2020). Venta a granel de embutidos: una tendencia de comercialización asociada al riesgo de enfermedades trasmitidas por alimentos en Culiacán, México. Rev Mex Cienc Pecu.

[2] Van Doren JM, Neil KP, Parish M, Gieraltowski L, Gould LH, Gombas KL (2013). Foodborne illness outbreaks from microbial contaminants in spices, 1973-2010. Food Microbiol.

[3] Drudge C, Greco S, Kim J, Copes R (2019). Estimated annual deaths, hospitalizations, and emergency department and physician office visits from foodborne illness in Ontario. Foodborne Pathog Dis.

[4] Rincón D, Ramírez R, Vargas J (2011). Transmisión de Salmonella entérica a través de huevos de gallina y su importancia en salud pública. Rev Univ Ind Santander Salud.

[5] Sevilla Ruiz MI, Franco Sánchez A (2015). Evolution of outbreaks caused by salmonella spp., in the Region of Murcia (2003-2013). Nutr Clín Diet Hosp.

[6] Molina-Alvarado A, Granados-Chinchilla F (2015). Inocuidad microbiológica de los alimentos para animales en Costa Rica. Nutr Anim Trop.

[7] Ventura-Ramón GH, Bueno-Durán AY, Toledo-Ibarra GA, Díaz-Resendiz KJG, Barcelos-García RG, Girón-Pérez MI (2020). Detección de Salmonella spp. en carne bovina procedente de rastros tipo inspección federal (TIF) y rastros "No-TIF" en Nayarit, México. Rev Bio Ciencias.

[8] Sheth AN, Hoekstra M, Patel N, Ewald G, Lord C, Clarke C (2011). A National outbreak of salmonella serotype Tennessee infections from contaminated peanut butter: a new food vehicle for salmonellosis in the United States. Clin Infect Dis.

[9] Werber D, Dreesman J, Feil F, van Treeck U, Fell G, Ethelberg S (2005). International outbreak of SalmonellaOranienburg due to German chocolate. BMC Infect Dis.

[10] Ríos K (2015). Implementación de pruebas de PCR para el diagnóstico sertotipo-especifico de Salmonella entérica serotipos Hadar y Typhimurium.

[11] Kérouanton A, Hennekinne JA, Letertre C, Petit L, Chesneau O, Brisa-bois A (2007). Characterization of Staphylococcus aureus strains associated with food poisoning outbreaks in France. Int JFood Microbiol.

[12] López GL, Alfonso, Suárez H (2016). Caracterización microbiológica y molecular de Staphylococcus aureus en productos cárnicos comercializados en Cartagena Colombia. Rev Costarric Salud Publica.

[13] Torres Vitela MR, Navarro Hidalgo V, Villarruel López A, Olea Rodríguez MA (2011). Prevalence of Salmonella and Staphylococcus aureus in chorizo and longaniza. Nacameh.

[14] Riquelme L (2007). Staphyloccus aureus en platos fríos listos para consumo en locales de comida italiana y medidas para su control.

[15] Martín Juárez B (2005). Estudio de las comunidades microbianas de embutidos fermentados ligeramente acidificados mediante técnicas moleculares estandarización, seguridad y mejora tecnológica.

[16] Salina M, Scholz L, Servián N, Romero M, Samudio T, Ruiz V (2018). Staphylococcus Aureus in food manipulators of gastronomic services of Asunción, Paraguay. Rev Salud Pública Paraguay.

[17] Jordá G, Marucci R, Guida A, Pires P, Manfredi E (2012). Portación y caracterización de Staphylococcus aureus en manipuladores de alimentos. Rev Argent Microbiol.

[18] Perlera de Escalante AE, Nishino S (2015). Determinación de la presencia de Salmonella spp; Staphylococcus aureus; Campylobacter jejuni y Campylobacter coli en productos cárnicos procesados en Cabañas y Cuscatlán. Prod Agropecu Desarro Sost.

[19] Cervantes-García E, García-González R, María Salazar-Schettino P (2014). Características generales del Staphylococcus aureus. Rev Latinoam Patol Clin Med Lab.

[20] Marzocca MA, Marucci PL, Sica MG, Álvarez EE (2004). Detección de Listeria monocytogenes en distintos productos alimenticios y en muestras ambientales de una amplia cadena de supermercados de la ciudad de Bahía Blanca (Argentina). Rev Argent Microbiol.

[21] Tzoc Ramírez E, Pineda S (2015). Aislamiento de Listeria monocytogenes en productos lácteos, artesanales y hortalizas. Rev Cienc Tecnol.

[22] Ortiz M (2016). Diversidad genética y persistencia ambiental de Listeria monocytogenes en dos plantas de procesado de carne de cerdo ibérico: influencia de la resistencia a desinfectantes de amonio cuaternario.

[23] López-Salazar J, Rodríguez-Haro C, López-Ullauri V, Díaz-Monroy B (2022). Prevalence of Listeria monocytogenes in fresh artisanal cheeses sold in public markets in the city of Riobamba, Ecuador. Rev Argent Microbiol.

[24] Chaves C, Arias ML (2009). Caracterización de cepas de Listeria monocytogenes realizados a partir de queso fresco proveniente de diferentes zonas productoras costarricenses. Arch Latinoam Nutr.

[25] Siriken B, Ayaz ND, Erol I (2014). Listeria monocytogenes in retailed raw chicken meat in Turkey. Berl Munch Tierarztl Wochenschr.

[26] Ramírez L, Morón de Salim A, Alfieri A, Gamboa O (2009). Frecuencia de Listeria monocytogenes en muestras de tomates (Lycopersicum esculentum) y cilantro (Coriandrum sativum) frescos en tres supermercados de Valencia, Venezuela. Arch Latinoam Nutr.

[27] Muñoz AI, Rodríguez EC (2021). Distribución y caracterización fenotípica y genotípica de Listeria monocytogenes en aislamientos de alimentos, Colombia, 2010-2018. Biomédica.

[28] Ledermann W (2019). Thomas Mann y las enfermedades infecciosas en la primera mitad del siglo XX. Parte II: Tuberculosis, cólera, trasplantes. Revista Chil Infect.

[29] Ulloa MT, Sanhueza C, Henríquez T, Aguayo B, Hermosilla G, Porte L (2019). Cepas chilenas de origen clínico de Vibrio cholerae no-O1, no-O139 portan los genes vcsN2, vcsC2, vcsV2, vspD, toxR2 y vopF del sistema de secreción T3SS2 presentes en una isla de patogenicidad. Rev Chil Infect.

[30] Blanco C (2008). Vibrios de mayor relevancia en los productos de la pesca.

[31] Rojas N, Muñoz G, Gárate L, Gonzalez D, Del Pozo M (2013). Aislamiento microbiológico de Vibrio cholerae y Vibrio parahaemolyticus en muestras de camarón coctelero en la ciudad de Puebla. Rev Enferm Infecc Microbiol.

[32] Olea A, Gonzalez C, Chiu M, Vallebuona C, Labraña M, Martiniello F (2005). Brote de gastroenteritis por Vibrio parahaemolyticus en Chile. Rev Chil Salud Publica.

[33] Liu J, Bai L, Li W, Han H, Fu P, Ma X (2018). Trends of foodborne diseases in China: lessons from laboratory-based surveillance since 2011. Front Med.

[34] Sánchez-Chica MIA JA, Correa-Ochoa MM, Aceves-Díez ÁE, Castañeda-Sandoval LM (2014). Direct detection of toxigenic genes of Bacillus cereus in corn starch and wheat flour using multiplex polymerase chain reaction (mPCR). Medicina Laboratorio.

[35] Dierick K, Van Coillie E, Swiecicka I, Meyfroidt G, Devlieger H, Meulemans A (2005). Fatal Family Outbreak of Bacillus cereus-Associated Food Poisoning. J Clin Microbiol.

[36] Tewari A, Abdullah S (2015). Bacillus cereus food poisoning: international and Indian perspective. J Food Sci Technol.

[37] Bennett SD, Walsh KA, Gould LH (2013). Foodborne disease outbreaks caused by Bacillus cereus, Clostridium perfringens, and Staphylococcus aureus--United States, 1998-2008. Clin Infect Dis.

[38] Alaridi N (2022). Risk of Bacillus cereus contamination in cooked rice. Food Sci Technol.

[39] Tejeda-Trujillo F, Villagrán-Padilla C, León-TeIIo G, Tejeda-Hernández M (2013). Investigación de Bacillus cereus y calidad sanitaria de muestras de arroz cocidos recolectadas en diferentes establecimientos de la ciudad de Puebla, México. 2013. CienciaUAT.

[40] Cortés-Sánchez ADJ, Díaz-Ramírez M, Guzmán-Medina CA (2018). Sobre Bacillus cereus y la inocuidad de los alimentos (una revisión). Rev Ciencias.

